# The complete chloroplast genome sequence of *Malva pusilla* Sm. (Malvaceae), a medicinal plant

**DOI:** 10.1080/23802359.2026.2658955

**Published:** 2026-05-18

**Authors:** Junjie Wei, Wenjing Li, Xingchen Liu, Yingying Yan, Xiaoming Guo, Peiyan Ai, Menghao Wang, Xiaolong Xie, Xiaozheng Luo, Jianfeng Chang, Yanan Cao, Lianzhen Li

**Affiliations:** a College of Agronomy, Henan Agricultural University, Zhengzhou, China; b College of Life Sciences, Henan Agricultural University, Zhengzhou, China; c College of Plant Protection, Henan Agricultural University, Zhengzhou, China; d School of Pharmacy, Henan University of Chinese Medicine, Zhengzhou, China

**Keywords:** *Malva pusilla*, plastome, phylogenetic analysis

## Abstract

*Malva pusilla* Sm. is a perennial herb used for both medicinal and edible purposes. Here, we sequenced and assembled the complete chloroplast genome (plastome) of *M. pusilla*. The plastome is 158,345 base pairs (bp) in length, with a GC content of 37.12%, and exhibits a typical quadripartite structure. A total of 129 genes were annotated, including 84 protein-coding genes (PCGs), 37 transfer RNA (tRNA) genes, and eight ribosomal RNA (rRNA) genes. Phylogenetic analysis based on complete plastome sequences supported the monophyly of *Malva*. These data provide a valuable genomic resource for the genus and support future phylogenetic and evolutionary studies.

## Introduction

*Malva pusilla* Sm. (1794), a perennial herb in the family Malvaceae, is widely distributed in Kazakhstan, Kyrgyzstan, Mongolia, Turkmenistan, Uzbekistan, and parts of Asia and Europe. In China, it occurs in Anhui, Gansu, Guizhou, Hebei, Henan, Jiangsu, Shaanxi, Shandong, Shanxi, Sichuan, Xinjiang, Xizang, and Yunnan provinces (Wu et al. 2007). Commonly known as round-leaved mallow (synonym: *Malva rotundifolia* L.), it is characterized by reniform leaves and a prominent taproot. The taproot is used in traditional Chinese medicine to replenish qi, stop sweating, promote diuresis and lactation, and resolve boils (Editorial Committee of Chinese Materia Medica 1999), with applications analogous to those of Astragali Radix (China Pharmacopoeia Committee 2025). Phytochemical studies have shown that its extracts are nutrient-rich (Devrim-Lanpir et al. 2023) and contain bioactive compounds including palmitic acid, β-sitosterol, sterol derivatives, phthalates, glycosides, and fatty acids such as oleic and stearic acids (Curts and Harris 1949; Lahloub et al. 2017; Devrim-Lanpir et al. 2023). Pharmacological studies have demonstrated antioxidant activity (Ganaie et al. 2017) and potential anti-allergic effects (Lahloub et al. 2017). Despite these findings, genomic information on *M. pusilla* remains limited.

Plastomes are widely used for phylogenetic reconstruction and molecular identification in angiosperms (Jansen et al. 2005; Jansen and Ruhlman 2012). The genus *Malva* comprises approximately 30 species (Wu et al. 2007), but complete plastomes have been reported for only four species (Abdullah et al. 2021; García-Mir et al. 2021; Li et al. 2020; Wang et al. 2020; Zhang et al. 2025). As of 1 December 2025, eleven accessions representing nine *Malva* species were available in National Center for Biotechnology Information (NCBI; https://www.ncbi.nlm.nih.gov/). However, the complete plastome of *M. pusilla* has not yet been reported, and its phylogenetic position based on whole-plastome data remains unresolved. Previous plastome-based studies in *Malva* were limited by insufficient taxon sampling (Maurya et al. 2025; Zhang et al. 2025). Here, we assembled and annotated the complete plastome of *M. pusilla* and reconstructed a plastome-based phylogeny including this species. This study provides the first complete plastome of *M. pusilla* and offers new insights into phylogenetic relationships within *Malva*.

## Materials and methods

**Figure 1.**
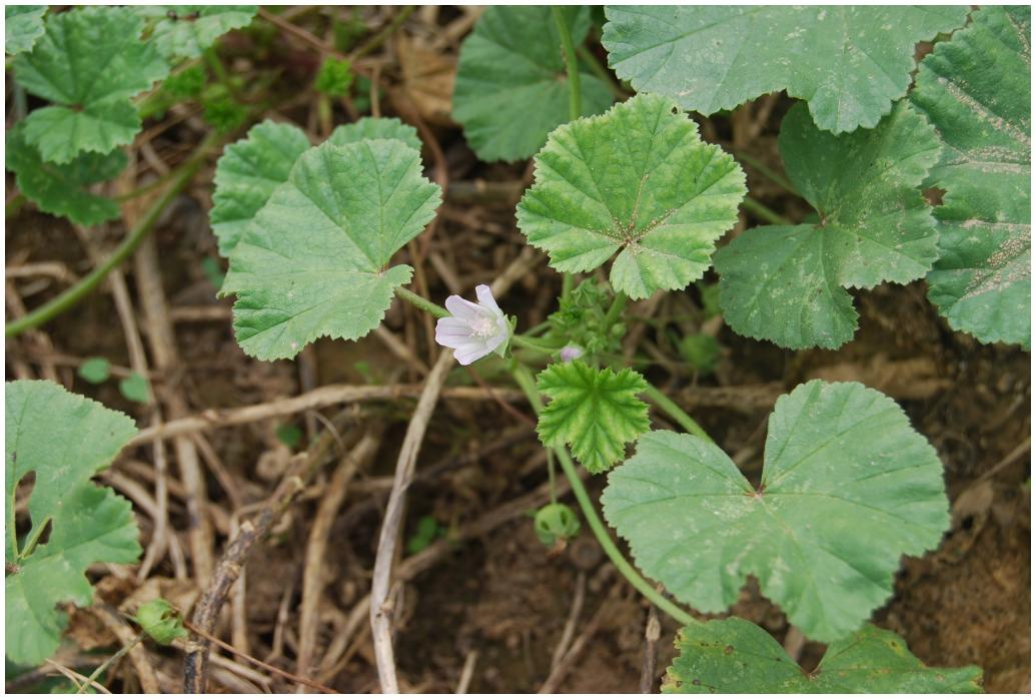
Morphological characteristics of *Malva pusilla*. *M. pusilla* is a perennial herb, typically characterized by reniform leaves with cordate base and serrated edges. Its small flower is white to pinkish with five petals. Photograph taken by Xiaolong Xie in Zhengzhou, China.

Fresh leaves were collected from Boxueyuan, Zhengdong New District, Zhengzhou, China (34.8196°N, 113.7919°E). The plant was subsequently transplanted to the medicinal botanical garden at the Longzi Lake campus of Henan Agricultural University, Zhengzhou, China. The specimen was identified as *Malva pusilla* Sm. by Profs. Xiaolong Xie and Xiaozheng Luo. A voucher specimen (JN20250815001) was deposited in the Herbarium of the College of Agronomy, Henan Agricultural University (contact: Junjie Wei; email: JunjieNgai2020@henau.edu.cn; Figure 1).

Total genomic DNA was extracted using the CTAB method (Doyle and Doyle 1987). Sequencing libraries were prepared, and paired-end sequencing (PE150) was performed on the DNBSEQ-T7 platform by Bena Technology Co. Ltd. (Wuhan, China). The plastome was assembled using GetOrganelle v1.7.7.1 (Jin et al. 2020) and visualized with Bandage v0.8.1 (Wick et al. 2015). Annotation was conducted using PGA (Qu et al. 2019), with *M. wigandii* plastome as a reference, followed by manual curation in Geneious Prime v2025.0.2 (Kearse et al. 2012). A circular genome map was generated using OGDRAW (Greiner et al. 2019).

For phylogenetic analysis, complete plastome sequences of 11 accessions representing nine additional *Malva* species were retrieved from NCBI (Table S1). Sequences were aligned using MAFFT v7.505 (Katoh et al. 2002) with the ‘–auto’ strategy. The orientations of the SSC and LSC regions were adjusted to match *M. pusilla* (Hu et al. 2025). A maximum-likelihood (ML) tree was constructed using IQ-TREE in PhyloSuite v1.2.2 (Zhang et al. 2020) under the best-fit model K3Pu + F + I with 1000 bootstrap replicates. *Althaea officinalis* and *Alcea rosea* were used as outgroups.

## Results

**Figure 2.**
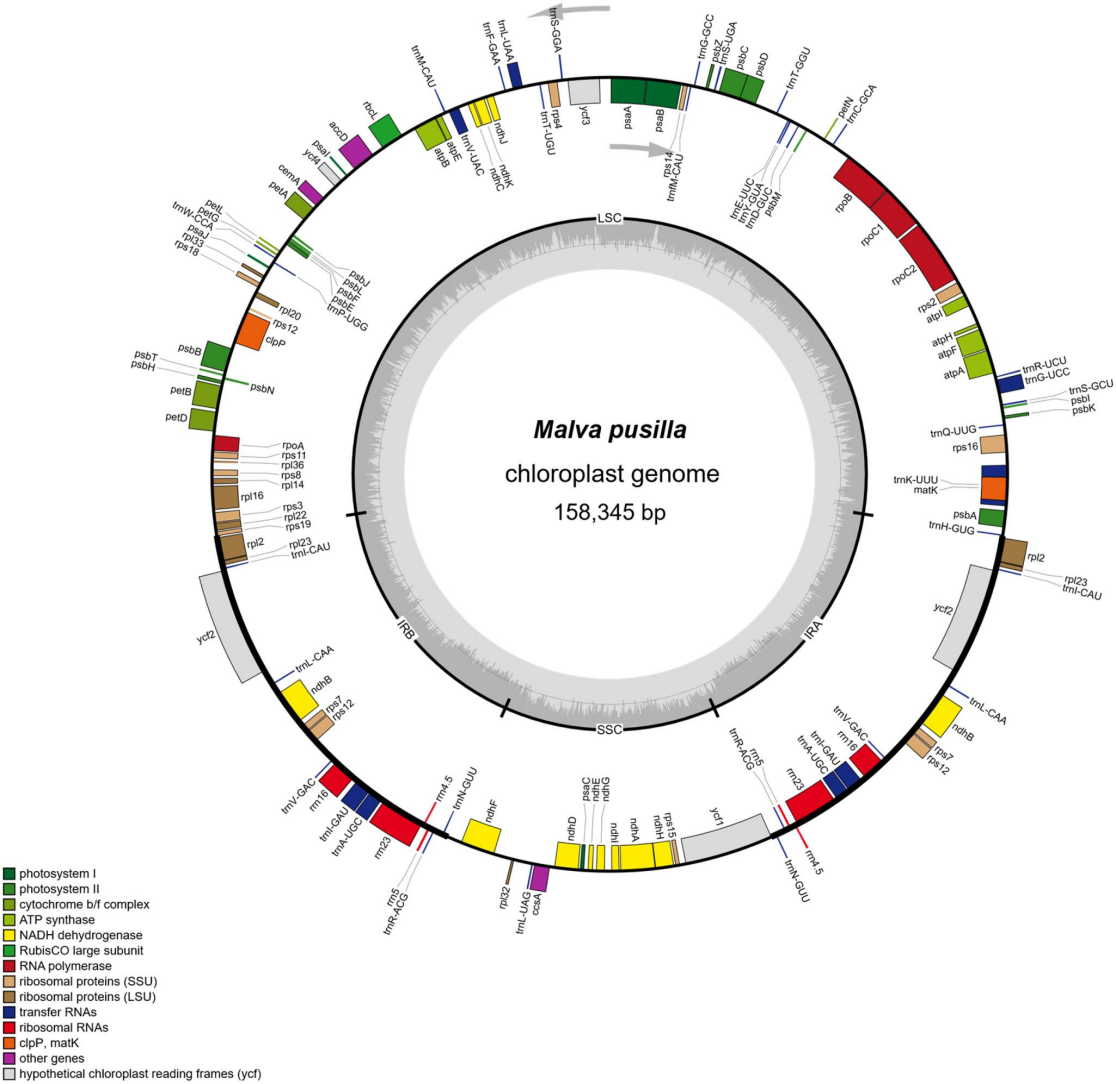
The complete plastome map of *Malva pusilla*. This circular diagram depicts the chloroplast genome of *M. pusilla*, with a total length of 158,345 bp. Colored blocks on the outer circle represent different functional gene classes, including photosystems I and II, ATP synthase, and ribosomal proteins. Genes located on the inner side of the outer circle are transcribed clockwise, as indicated by grey arrows, while those on the outer side are transcribed counterclockwise. The four structural regions—Large Single Copy (LSC), Small Single Copy (SSC), and Inverted Repeats (IRA and IRB)—are clearly annotated. The inner circle displays the GC and AT content variation across the genome, with GC content indicated by dark grey shading while AT content by light grey shading.

The plastome of *M. pusilla* (GenBank: PX552231.1) is 158,345 bp in length and displays a typical quadripartite structure, comprising an LSC region (87,051 bp), an SSC region (21,104 bp), and two IR regions (25,095 bp each). The overall GC content is 37.12%, with regional values of 34.92% (LSC), 32.12% (SSC), and 43.02% (IRs). Sequencing coverage ranged from 888× to 9555×, with an average of 6,023× (Figure S1). The plastome contains 129 annotated genes, including 84 PCGs, 37 tRNA genes, and eight rRNA genes (Figure 2). Seventeen genes are duplicated in the IR regions, including six PCGs (*ndhB*, *rpl2*, *rpl23*, *rps7*, *rps12*, and *ycf2*), seven tRNA genes (*trnA-UGC*, *trnI-CAU*, *trnI-GAU*, *trnL-CAA*, *trnN-GUU*, *trnR-ACG*, and *trnV-GAC*), and four rRNA genes (*rrn5*, *rrn16*, *rrn4.5*, and *rrn23*). Three genes (*clpP*, *rps12*, and *ycf3*) contain two introns each, whereas nine PCGs (*ndhA*, *ndhB*, *petB*, *petD*, *atpF*, *rpl2*, *rpl16*, *rps16*, and *rpoC1*) and six tRNA genes (*trnA-UGC*, *trnG-UCC*, *trnI-GAU*, *trnK-UUU*, *trnL-UAA*, *trnV-UAC*) contain a single intron. Additionally, eleven genes (*rps16*, *atpF*, *rpoC1*, *ycf3*, *clpP*, *petB*, *petD*, *rpl16*, *rpl2*, *ndhB*, and *ndhA*) are cis-spliced, and the *rps12* gene is trans-spliced (Figures S2 and S3).

**Figure 3.**
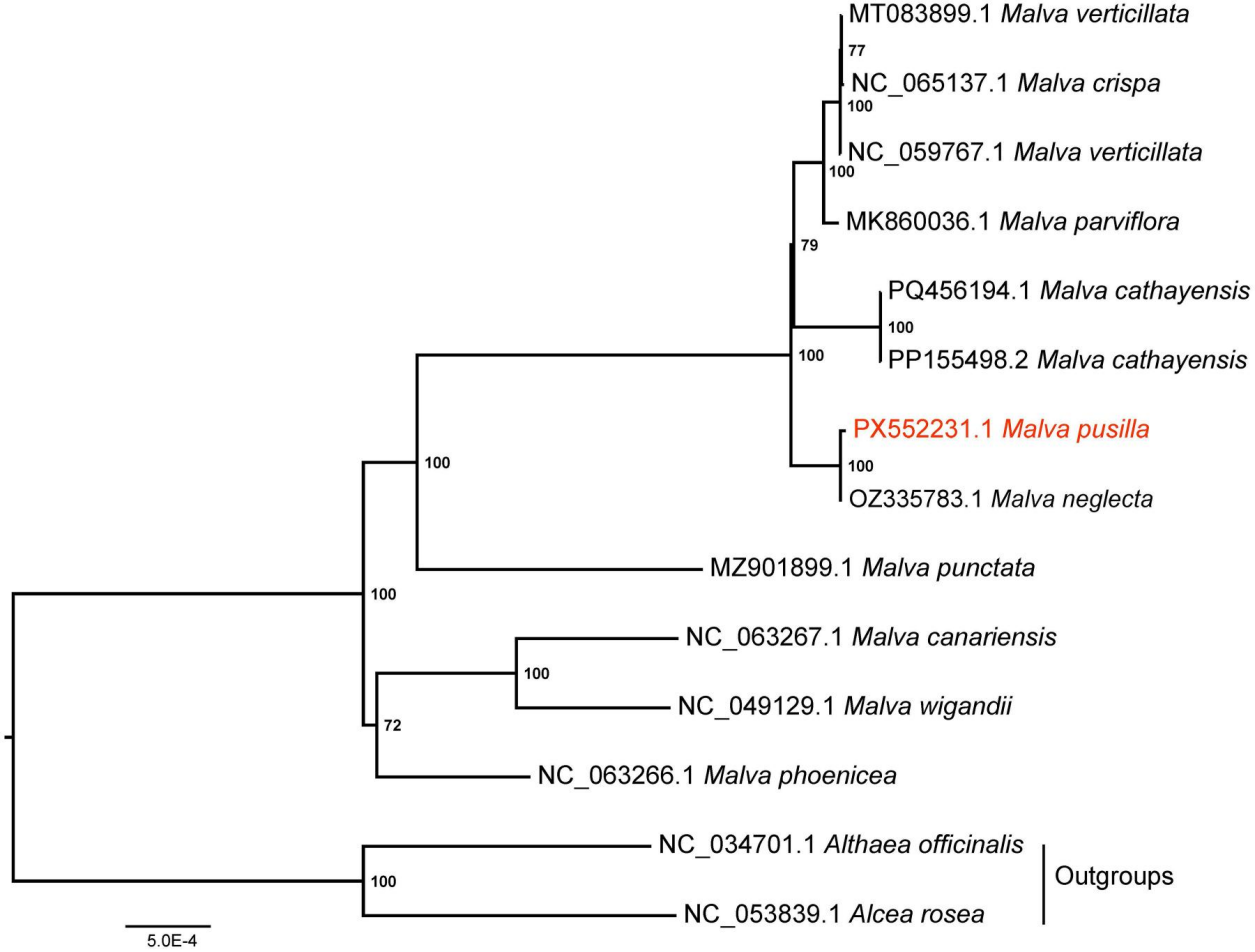
Phylogenetic relationships between *Malva pusilla* and other representative species of the genus *Malva*. This figure displays a phylogenetic tree illustrating the relationships among 10 *Malva* species (12 accessions) and two outgroup taxa, *Althaea officinalis* and *Alcea rosea*. Phylogenetic lineages are represented by distinct branches, with bootstrap support values labeled at corresponding nodes. *M. pusilla* is prominently highlighted in red. A scale bar at the bottom indicates a genetic distance of 5.0E-4. GenBank accession numbers and corresponding references for the sequences included in the analysis are as follows: *M. verticillata* (NC_059767.1, Li et al. 2020; MT083899.1, Wang et al. 2020), *M. crispa* (NC_065137.1), *M. parviflora* (MK860036.1, Abdullah et al. 2021), *M. cathayensis* (PP155498.2, Zhang et al. 2025; PQ456194.1), *M. neglecta* (OZ335783.1), *M. punctata* (MZ901899.1), *M. canariensis* (NC_063267.1), *M. pusilla* (PX552231.1, this study), *M. wigandii* (NC_049129.1, García-Mir et al. 2021), *M. phoenicea* (NC_063266.1), *Althaea officinalis* (NC_034701.1, Zhang et al. 2017), and *Alcea rosea* (NC_053839.1, Qian et al. 2020).

Phylogenetic analysis supported the monophyly of *Malva*, with all species forming a well-supported clade (Figure 3). Two subclades were resolved: one including *M. wigandii*, *M. canariensis*, and *M. phoenicea*, and the other including *M. pusilla* and six additional species. Within the latter clade, *M. pusilla* was resolved as sister to *M. neglecta*.

## Discussion and conclusion

We report the complete plastome of *M. pusilla*, which exhibits a typical angiosperm plastome structure, consistent with previous observations (Jansen et al. 2005; Jansen and Ruhlman 2012). Comparison with other *Malva* plastomes indicates high structural conservation, with genome sizes ranging from 158,162 to 158,793 bp and gene numbers from 129 to 131 (Table S1). This pattern is consistent with the generally conserved nature of plastomes in angiosperms.

Previous studies have generally recognized two major clades within *Malva* (Dalby 1968; Ray 1995, 1998; Jędrzejczyk and Rewers 2020; Maurya et al. 2025; Zhang et al. 2025; Zhou et al. 2025). Based on complete plastome data from six species (seven accessions), Zhang et al. (2025) identified one clade comprising *M. wigandii* and *M. canariensis* (Clade I), whereas the other comprised *M. cathayensis*, *M. crispa*, *M. verticillata*, and *M. parviflora* (Clade II). Our plastome-based phylogeny recovered a similar topology with expanded taxon sampling, thereby improving resolution within the genus. One clade corresponds to Clade I but additionally includes *M. phoenicea*, whereas the other resembles Clade II but additionally includes *M. punctata*, *M. neglecta*, and *M. pusilla*. Within this clade, *M. pusilla* was resolved as sister to *M. neglecta*. Notably, accessions MZ901899.1 (labeled as *Lavatera punctata*) and NC_063266.1 (labeled as *Navaea phoenicea*) are currently treated as synonyms of *Malva punctata* and *Malva phoenicea* in Plants of the World Online (POWO; https://powo.science.kew.org/) and Global Biodiversity Information Facility (GBIF; https://www.gbif.org/), respectively. Their placement within *Malva* in our phylogeny is consistent with these taxonomic treatments.

In summary, the plastome of *M. pusilla* provides a valuable genomic resource and contributes to resolving phylogenetic relationships within *Malva*. The plastome-based phylogeny clarifies the molecular position of *M. pusilla* and improves understanding of relationships among closely related species.

## Supplementary Material

Supplementary Material_for review.docx

## Data Availability

The genome sequence data that support the findings of this study are openly available in GenBank of NCBI at https://www.ncbi.nlm.nih.gov/ under accession no. PX552231.1. The associated BioProject, Bio-Sample and SRA numbers are PRJNA1401681, SAMN54568379 and SRR36784097, respectively.
