# Supplementary material for: The complete chloroplast genome sequence of Malva pusilla Sm. (Malvaceae), a medicinal plant
Source: Mitochondrial DNA B Resour. 2026 May 18;11(6):750–4. doi: 10.1080/23802359.2026.2658955 (PMC13185054; doi:10.1080/23802359.2026.2658955)
Supplement: Supplementary Material_for review.docx [file TMDN_A_2658955_SM8114.docx]

**Supplementary materials**

**
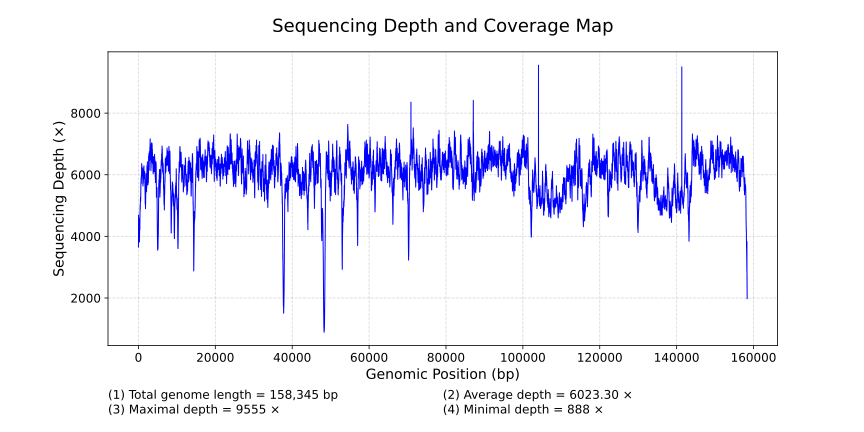
**

**Figure S1.** **Sequencing depth and coverage map of the *Malva pusilla* chloroplast genome.** The sequencing reads coverage and depth of *M. pusilla* chloroplast genome is shown in this figure. The minimal, maximal and average read mapping depths were 888×, 9555× and 6023×, respectively. Data analysis and figure was prepared according to the method in Ni et al. (2023).


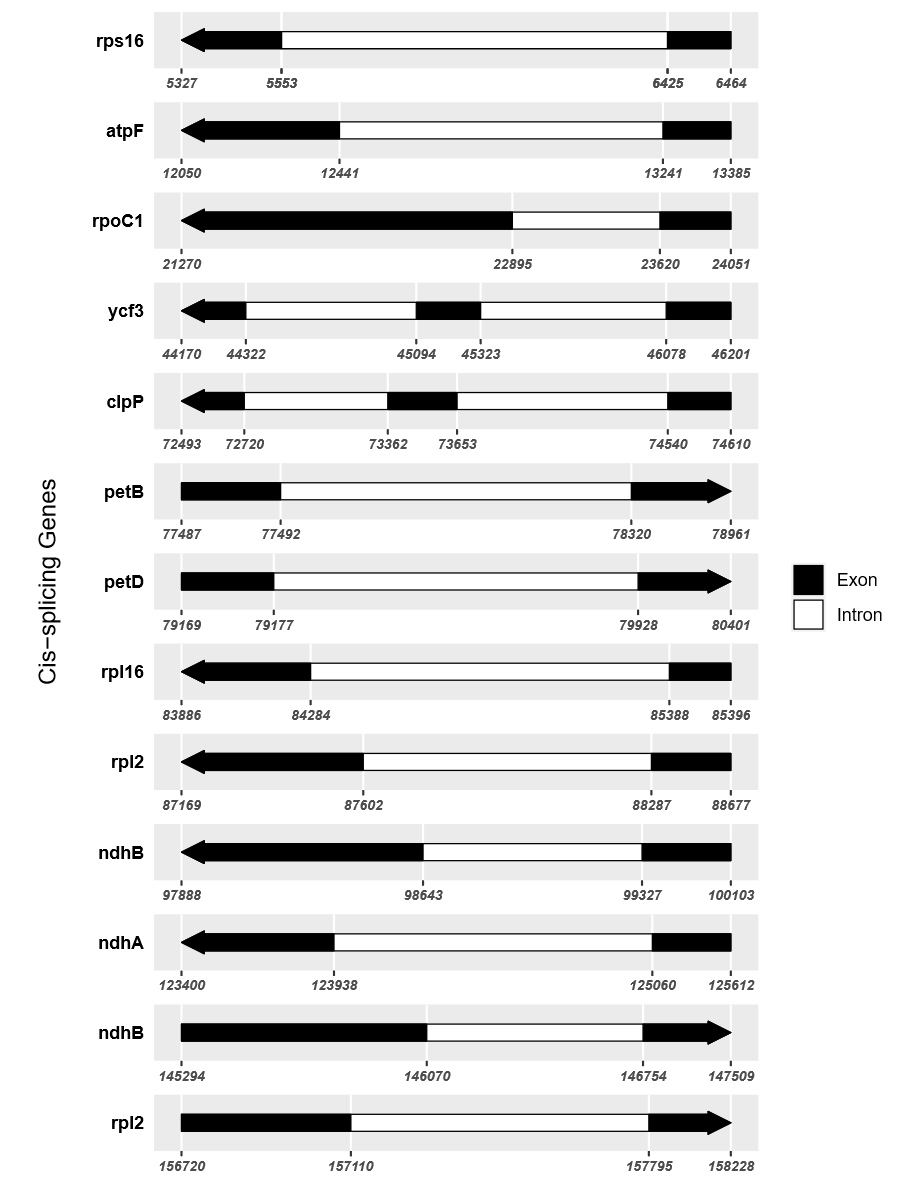


**Figure S2.** **Schematic map of the cis-splicing genes in the** **chloroplast genome of *M. pusilla*.** Exons and introns are shown in black and white separately, while arrows indicate the sense direction of transcription. The numbers below each schematic map demonstrate the location in the chloroplast genome. This map was not drawn to scale with CPGView (Liu et al. 2023).


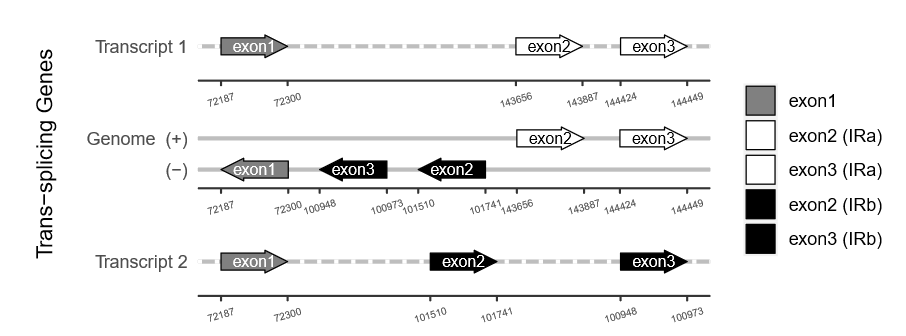


**Figure S3.** **Schematic map of the trans-splicing gene *rps12* in the chloroplast genome of *M. pusilla*.** The arrows of exons indicate the sense direction of transcription. The numbers below each schematic map represent the location in the chloroplast genome*.* This map was not drawn to scale with CPGView (Liu et al. 2023).

**References**

Liu S, Ni Y, Li J, Zhang X, Yang H, Chen H, liu C. 2023. CPGView: a package for visualizing detailed chloroplast genome structures. Molecular Ecology Resources. 23(3): 694-704. doi: 10.1111/1755-0998.13729.

Ni Y, Li JL, Zhang C, Liu C. 2023. Generating sequencing depth and coverage map for organelle genomes. protocols.io. doi: dx.doi.org/10.17504/protocols.io.4r3l27jkxg1y/ v1.
